# Determinants of Metabolic Dysfunction-Associated Steatotic Liver Diseases in Patients with Type 2 Diabetes Mellitus

**DOI:** 10.3390/jcm15166421

**Published:** 2026-08-19

**Authors:** Monica Potra, Simona Popescu, Bogdan Timar, Sandra Lazar, Laura Gaita, Adina Braha, Teodora Sorescu, Laura Diaconu, Liana Iordan, Andreea Papoi, Adriana Ivanescu, Romulus Timar

**Affiliations:** 1 Doctoral School of Medicine, “Victor Babes” University of Medicine and Pharmacy, 300041 Timisoara, Romania; monica.rusu@umft.ro (M.P.);; 2 Department of Diabetes, Nutrition, and Metabolic Diseases Clinic, Arad County Clinical Hospital, 310037 Arad, Romania; 3 Center for Molecular Research in Nephrology and Vascular Disease, “Victor Babes” University of Medicine and Pharmacy, 300041 Timisoara, Romaniasorescu.teodora@umft.ro (T.S.); diaconu.laura@umft.ro (L.D.); timar.romulus@umft.ro (R.T.); 4 Department of Diabetes, “Pius Brinzeu” Emergency Hospital, 300736 Timisoara, Romania; 5 Second Department of Internal Medicine, “Victor Babes” University of Medicine and Pharmacy, 300041 Timisoara, Romania; 6 Department of Hematology, Emergency Municipal Hospital, 300254 Timisoara, Romania; 7 First Department of Internal Medicine, “Victor Babes” University of Medicine and Pharmacy, 300041 Timisoara, Romania; 8 Department of Gastroenterology, Arad County Clinical Hospital, 310037 Arad, Romania; papoi.andreea@uvvg.ro; 9 Opticlass Ophthalmology Clinic, 30012 Timisoara, Romania

**Keywords:** diabetes mellitus, MASLD, risk factors

## Abstract

**Background:** Type 2 diabetes mellitus (T2DM) is one of the most complex metabolic disorders worldwide, with a continuously rising prevalence. T2DM is linked to multiple complications, among which metabolic dysfunction-associated steatotic liver disease (MASLD) has gained increasing recognition. MASLD is the leading cause of chronic liver disease and a major determinant of cirrhosis and hepatocellular carcinoma. This research aimed to estimate the prevalence of MASLD in a single-center outpatient diabetes clinic and explore the associated risk factors. **Methods:** The study included 170 adults previously diagnosed with T2DM. Data regarding disease duration, metabolic control, demographic characteristics, anthropometric parameters, and comorbidities were retrieved. Laboratory analyses and imaging investigations were conducted to assess liver status and MASLD presence. **Results:** The prevalence of MASLD was 76.47%. Patients with MASLD presented significantly higher body weight, waist circumference, and body mass index (all *p* < 0.0001). The MASLD population exhibited longer diabetes duration (*p* = 0.043) and poor metabolic control. The lipid profile showed higher LDL-cholesterol (*p* = 0.003) and triglycerides (*p* < 0.0001) and lower HDL-cholesterol (*p* = 0.0004). Insulin resistance indices illustrated significant differences, with higher METS-IR and lower eGDR in MASLD patients (both *p* < 0.0001). **Conclusions:** MASLD was highly prevalent in the evaluated population. Our findings suggest that MASLD occurrence is mainly associated with obesity, insulin resistance, inadequate glycemic control, and dyslipidemia. All these factors indicate an unfavorable metabolic profile in T2DM patients, underscoring the need for early liver function screening.

## 1. Introduction

Type 2 diabetes mellitus (T2DM) is a highly prevalent and complex metabolic disorder associated with multiple complications that pose a substantial public health burden [1]. Among the comorbidities frequently associated with T2DM, metabolic dysfunction-associated steatotic liver disease (MASLD) has gained increasing recognition [2]. MASLD is defined by the presence of hepatic steatosis in patients with cardiometabolic risk factors and, by definition, excludes alternative causes of liver fat accumulation, such as excessive alcohol intake or viral hepatitis [3]. MASLD is the primary cause of chronic liver disease worldwide and is also emerging as a leading cause of cirrhosis and hepatocellular carcinoma [1,4].

MASLD and T2DM share common pathogenic mechanisms, with insulin resistance (IR) representing a central driver of MASLD pathophysiology [5]. In addition to IR, a significant component of MASLD pathophysiology is represented by chronic low-grade metabolic inflammation. The release of pro-inflammatory mediators in conjunction with adipose tissue dysfunction contributes to hepatic lipid accumulation, cellular stress, and liver injury, linking systemic metabolic dysfunction to hepatic disease [6]. The connection between T2DM and MASLD is strong and bidirectional: T2DM significantly influences the development and progression of MASLD, while individuals with MASLD are at increased risk of developing T2DM and of experiencing T2DM-related complications, particularly cardiovascular disease and chronic kidney disease (CKD) [7]. Notably, approximately 65% of patients with T2DM are affected by MASLD [8], and the prevalence of advanced fibrosis (AF) in adults with T2DM treated in tertiary care centers has been reported to range from 14% to 38%, exceeding that observed in non-diabetic populations [9,10,11,12].

MASLD is currently the most widespread chronic liver pathology, affecting approximately one-third of the general adult population [8] and around 10% of children and adolescents [13]. Over recent decades, its prevalence has risen substantially, in parallel with the global increase in obesity and diabetes rates [14]. Except for genetically driven forms of the disease [15], MASLD is closely related to IR [15]; therefore, it is often perceived as the hepatic display of metabolic syndrome [16], a connection further supported by the markedly higher prevalence of MASLD among individuals with severe obesity and T2DM [17,18]. Emerging evidence indicates that IR contributes not only to the development of MASLD but also to its progression toward more advanced histological stages, including metabolic dysfunction-associated steatohepatitis and advanced liver fibrosis [19]. Notably, liver fibrosis is the strongest predictor of progression to cirrhosis and of clinically significant liver-related outcomes, such as hepatic decompensation and mortality [15,20].

IR represents a central pathophysiological process underlying numerous metabolic disorders [21,22], and substantial evidence identifies steatotic liver disease as the hepatic manifestation of systemic IR [23,24,25]. In MASLD development, IR both triggers and sustains a complex cascade characterized by dysglycemia, hyperinsulinemia, and intrahepatic triglyceride accumulation, thereby establishing a self-reinforcing cycle of metabolic dysfunction [26,27,28]. In addition, chronic metabolic inflammation may heighten hepatic injury through sustained pro-inflammatory signaling in conjunction with adipose tissue dysfunction and systemic metabolic abnormalities. Therefore, IR and metabolic inflammation are interconnected processes that contribute simultaneously to the hepatic manifestations of metabolic dysfunction and ultimately MASLD progression [29,30,31].

MASLD can progress to advanced liver disease and lead to significant hepatic complications [4]. In addition, it may promote systemic inflammation and exacerbate overall metabolic dysfunction [32]. Consequently, timely identification of MASLD in patients with T2DM is crucial for optimal disease management and targeted therapeutic strategies. Recent evidence highlights the value of prompt lifestyle modifications and pharmacological interventions in preventing or even reversing disease progression, particularly when MASLD is addressed early [4,33,34].

The primary objective of our study was to determine the prevalence of MASLD in individuals with T2DM and to identify key risk factors. We correlated MASLD with IR and other cardiovascular risk factors to determine cutoff values for increased hepatic steatosis risk.

## 2. Materials and Methods

### 2.1. Study Design and Population

This non-interventional, cross-sectional study was performed at the Outpatient Diabetes Care Center of County Emergency Hospital in Arad, Romania, between 16 July 2024 and 16 September 2024. Of the 243 adult patients who attended their preset visits at the diabetes center, we enlisted 170 participants with T2DM (89 females, 81 males). Of the total number of patients, 40 refused to participate in this study, and 33 did not fulfill the study’s eligibility criteria. Selected individuals were aged 18 years or older and had been previously diagnosed with T2DM. Exclusion criteria referred to severe cognitive impairment, psychiatric or neurological pathologies that prevented patients from providing informed consent, institutionalized patients, and other medical disorders that required hospitalization throughout the study. In addition, patients with a history of viral or autoimmune hepatitis, hepatic tumors, drug-induced liver disease (e.g., related to amiodarone, corticosteroids, tamoxifen, methotrexate, antiepileptics, antiretrovirals, oral contraceptives, statins, or potentially hepatotoxic supplements), hepatolenticular degeneration, excessive alcohol intake, or severe hepatic insufficiency were excluded. Participants were not implicated in the development of this study, and informed consent was attained from the entire research group. This study was approved by the Ethics Committee of the Emergency County Hospital, Arad (approval no. 130/19 May 2026), and was conducted in accordance with the Declaration of Helsinki (2013 version).

The study protocol included the evaluation of anthropometric and demographic characteristics, DM profile, metabolic control, and systemic comorbidities. In addition, laboratory analyses and specific imaging investigations were performed to assess liver status and the presence of MASLD.

### 2.2. Data Collection and Medical Assessment

The study protocol included a comprehensive assessment of demographic, anthropometric, clinical, metabolic, laboratory, and liver-related parameters. Demographic and clinical characteristics included gender, age, and diabetes duration, as well as anthropometric measurements (body weight, height, and body mass index [BMI]) and waist circumference (WC). Glycemic control was evaluated by measuring plasma glucose levels (both fasting and postprandial) as well as glycated hemoglobin (HbA1c). Laboratory evaluations included lipid profile assessment, comprising serum triglycerides, total cholesterol, low-density lipoprotein cholesterol (LDL-c), and high-density lipoprotein cholesterol (HDL-c).

Patients were systematically screened for diabetes-related complications and systemic comorbidities. Microvascular complications included peripheral neuropathy (PN), diabetic retinopathy, and chronic kidney disease. Macrovascular complications included ischemic heart disease and heart failure. Hypertension was assessed as a major cardiovascular comorbidity. Liver status was assessed using a combination of laboratory tests and imaging investigations. Biochemical analyses included alanine aminotransferase (ALT) and aspartate aminotransferase (AST). Abdominal ultrasonography and transient elastography (FibroScan, Echosens, 502 Touch, Paris, France; probe M and XL according to manufacturer guidelines) were performed by a certified operator in a fasting state. At least 10 valid measurements were obtained, with an interquartile range to median ratio (IQR/median) below 30%, in accordance with current recommendations to ensure the reliability of controlled attenuation parameter (CAP) and liver stiffness measurements. Liver fibrosis was categorized into four stages according to previously transient elastography thresholds: F0–F1, ≤8.2 kPa; F2, 8.3–9.7 kPa; F3, 9.8–13.6 kPa; and F4, >13.6 kPa [35,36]. Hepatic steatosis was quantified using CAP, with values expressed in decibels per meter (dB/m). Based on previously validated thresholds, steatosis was defined as CAP ≥248 dB/m and further stratified as mild (248–268 dB/m), moderate (269–279 dB/m), or severe (≥280 dB/m) [37]. In addition to elastography, liver-related fibrosis was further evaluated using established noninvasive serum-based indices. The Fibrosis-4 (FIB-4) index was calculated using the following formula: age (years) × AST (U/L)/[platelet count (10^9^/L) × √ALT (U/L)] [38]. Because age is a component of the FIB-4 formula and may lead to an overestimation of fibrosis risk in older adults, age-adjusted cutoffs were applied. In patients aged 60–65 years, FIB-4 values <1.3, 1.3–2.67, and >2.67 indicated low, indeterminate, and high probability of advanced fibrosis, respectively. In patients aged >65 years, the lower cutoff was increased to 2.0, with values <2.0, 2.0–2.67, and >2.67 indicating low, indeterminate, and high probability, respectively. These age-adjusted thresholds were applied in accordance with McPherson et al. and current recommendations from the American Association for the Study of Liver Diseases and the European Association for the Study of the Liver, European Association for the Study of Diabetes, and European Association for the Study of Obesity [39]. The Aspartate Aminotransferase to Platelet Ratio Index (APRI) was calculated as [(AST/upper limit of normal AST)/platelet count (10^9^/L)] × 100. These scores were used as noninvasive surrogate markers of hepatic fibrosis severity [40].

MASLD was defined as the presence of hepatic steatosis together with at least one of the following cardiometabolic risk factors [41]:

- BMI ≥ 25 kg/m^2^ or waist circumference > 94 cm in men and >80 cm in women; fasting plasma glucose ≥ 100 mg/dL, 2-h postprandial glucose ≥ 140 mg/dL, HbA1c ≥ 5.7%, or use of glucose-lowering medication;
- Systolic or diastolic blood pressure ≥ 130/85 mmHg or antihypertensive treatment;
- Serum triglycerides ≥ 150 mg/dL or treatment for hypertriglyceridemia;
- HDL-cholesterol <40 mg/dL in men or <50 mg/dL in women, or treatment for dyslipidemia.

As all participants had T2DM and therefore fulfilled at least one cardiometabolic risk criterion, MASLD classification in the present cohort was based on the presence of hepatic steatosis, defined as a CAP value ≥ 248 dB/m. Participants with CAP < 248 dB/m were classified as having non-MASLD.

IR was assessed using four surrogate indices, each calculated separately and analyzed independently:

1. Triglyceride–glucose (TyG) index: ln [fasting triglycerides (mg/dL) × fasting plasma glucose (mg/dL)/2];

2. Triglyceride to HDL-cholesterol ratio (TG/HDL-c): TG (mg/dL)/HDL-c (mg/dL);

3. Metabolic score for insulin resistance (METS-IR): ln[(2 × fasting blood glucose (mg/dL) + triglycerides (mg/dL))] × BMI (kg/m^2^)/ln[HDL-cholesterol (mg/dL)];

4. Estimated Glucose Disposal Rate (eGDR): 21,158 − (0.09 × WC) − (3.407 × HT) − (0.551 × HbA1c).

### 2.3. Statistical Analysis

Statistical analysis was performed using MedCalc^®^ version 23.3.7 (MedCalc Software Ltd., Ostend, Belgium).

The Shapiro–Wilk test was used to assess the normality of the variable distribution. Since the continuous variables were not normally distributed, results are reported as median values with 25th and 75th percentiles, and inter-group variations were evaluated using the Mann–Whitney U test. Categorical data are reported as frequencies and percentages. Categorical variables were compared across groups using either the chi-square or Fisher’s exact test, based on data characteristics.

Associations between CAP, indices of IR, and cardiovascular risk were assessed using Spearman’s rank correlation coefficient (ρ). Linear regression models were further applied to evaluate relationships between metabolic biomarkers and elevated hepatic steatosis risk. To ensure statistical robustness and to minimize multicollinearity, multivariable linear regression models were constructed separately by domain with a limited number of predictors (2–3 variables per model) rather than a single saturated model. Receiver operating characteristic (ROC) analysis was performed to determine the discriminative performance of inflammatory markers in distinguishing patients with MASLD from those without. Optimal cutoff points were calculated using Youden’s index (sensitivity + specificity − 1), with area under the curve (AUC) values and their corresponding 95% confidence intervals (CIs) generated to evaluate diagnostic performance across markers.

Statistical significance was defined as a two-sided *p*-value < 0.05.

## 3. Results

### 3.1. Study Population

Based on the defined selection criteria, 170 patients with T2DM were included. The median age of the group was 61 years (IQR: 56–67), and the median duration of diabetes was 5 years (IQR: 1–10). The study population consisted of 81 men (47.6%) and 89 women (52.4%). Glycemic control was generally suboptimal, with a median HbA1c of 7.2% (IQR: 6.8–8). A comparative analysis revealed no significant differences between male and female patients for most measured parameters. However, statistically significant differences in anthropometric parameters were identified. Men had higher body weight (*p* = 0.039), BMI (*p* = 0.029), and waist circumference (*p* = 0.005). Regarding glycemic parameters, females had slightly higher blood glucose levels (*p* = 0.045). Glycemic control was comparable between the two groups, with no significant sex-related differences in HbA1c (*p* = 0.189) or C-peptide levels (*p* = 0.738). The lipid profile, hepatic markers, and IR indices (METS-IR, TyG, TG/HDLc, and eGFR) were comparable between sexes (all *p* > 0.05). There were also no significant differences in hepatic steatosis (CAP), fibrosis indices (FIB-4 and APRI), or liver stiffness between the groups (Table 1).

### 3.2. Comparison of Metabolic Parameters According to MASLD Status

We compared the main clinical and laboratory parameters between patients with and without MASLD and identified significant differences across most variables. Table 2 presents the clinical, anthropometric, and biochemical characteristics of the participants, compared according to the presence of MASLD. Of the 170 patients, 23.5% (*n* = 40) did not have MASLD, whereas 76.5% (*n* = 130) were diagnosed with MASLD. No significant differences were observed between the groups with respect to sex distribution (men: 42.5% vs. 49.2%, *p* = 0.457) or median age (60 vs. 61.5 years, *p* = 0.273). In contrast, patients with MASLD had significantly higher body weight, waist circumference, and BMI (all *p* < 0.0001), resulting in a less favorable anthropometric profile. Diabetes duration was also longer in this group (*p* = 0.043).

Glycemic parameters were higher in patients with MASLD, including fasting plasma glucose (*p* = 0.009), HbA1c (*p* = 0.006), and C-peptide (*p* < 0.0001), consistent with reduced metabolic control. The lipid profile differed between groups, with higher LDL-cholesterol (*p* = 0.003) and triglycerides (*p* < 0.0001) and lower HDL-cholesterol (*p* = 0.0004) in the MASLD group.

Regarding liver function, AST levels were significantly elevated in MASLD patients (*p* = 0.003), while ALT concentrations remained comparable across groups (*p* = 0.867). IR indices showed marked differences: METS-IR was significantly higher and eGDR significantly lower in patients with MASLD (both *p* < 0.0001), consistent with greater IR.

**Figure 1:**
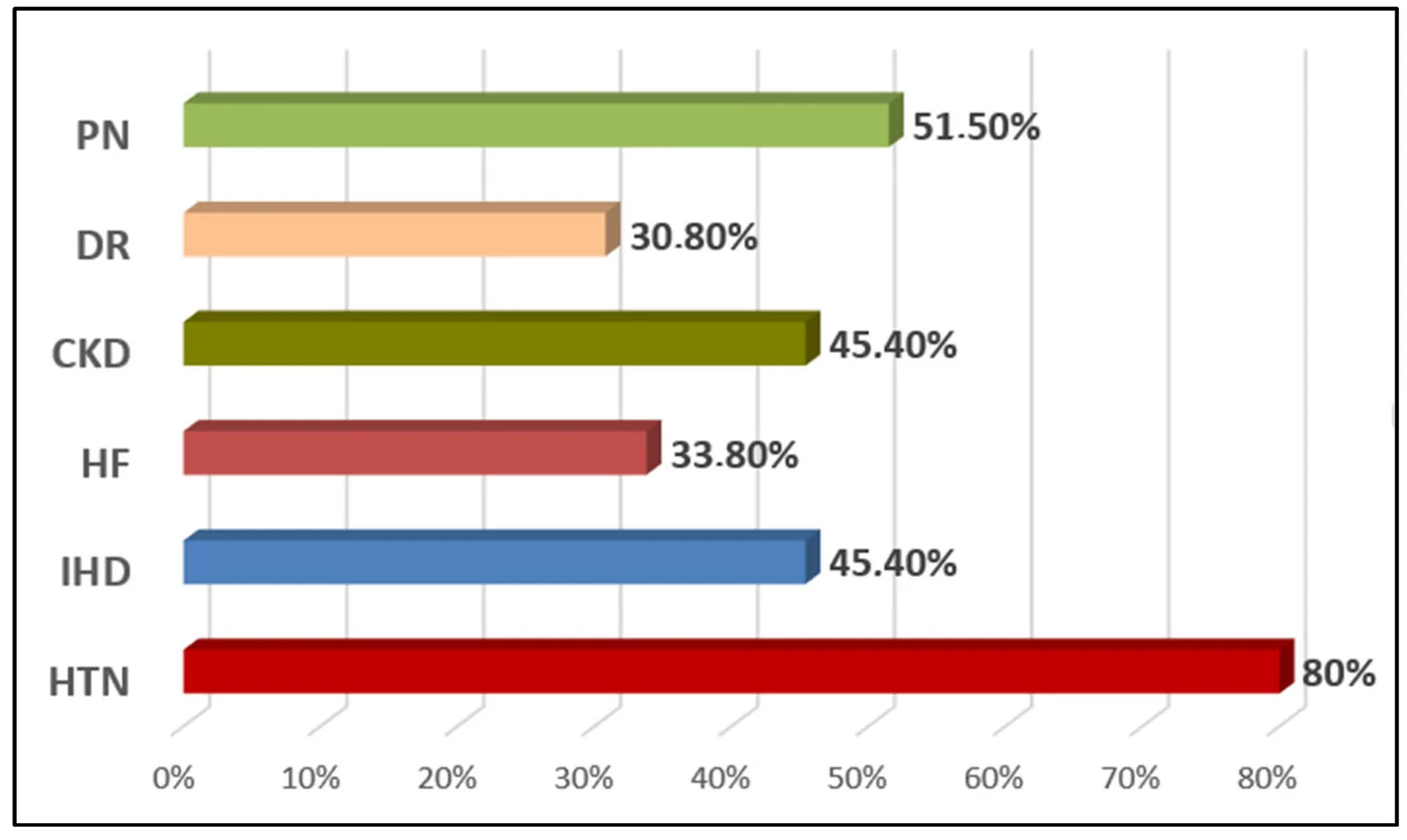
Prevalence of common comorbidities in studied patients with MASLD. Abbreviations: PN, peripheral neuropathy; DR, diabetic retinopathy; CKD, chronic kidney disease; HF, heart failure; IHD, ischemic heart disease; HTN, hypertension.

We also evaluated the prevalence of major diabetes-related complications within the study population. The most common comorbidities among patients with MASLD were hypertension (80%), followed by peripheral neuropathy (51.5%), ischemic heart disease (45.4%), and CKD (45.4%) (Figure 1).

### 3.3. Correlation Between Hepatic Steatosis (CAP) and Insulin Resistance Parameters

Spearman correlation analysis was performed to evaluate the association between CAP values and metabolic, anthropometric, and IR-related parameters. Diabetes duration showed the highest correlation with CAP (*ρ* = 0.578), followed by triglycerides (*ρ* = 0.403) and IR indices (TyG, *ρ* = 0.367; METS-IR, *ρ* = 0.325). In contrast, less significant associations were found for anthropometric and lipid parameters, as detailed in Table 3.

### 3.4. Predictors of Hepatic Steatosis (CAP) and Optimal Cutoff Values

Multivariable linear regression models were constructed separately by domain (anthropometric, metabolic, and insulin resistance parameters) to limit multicollinearity.

Based on the anthropometric model (Table 4), waist circumference was the primary predictor of CAP (β = 1.59, 95% CI: 1.06–2.13, *p* < 0.0001), followed by BMI (β = 1.83, 95% CI: 0.58–3.08, *p* = 0.0042) and age (β = 1.00, 95% CI: 0.24–1.76, *p* = 0.0106). The model explained 37.6% of CAP variability (R^2^ = 0.376, *p* < 0.0001).

In the metabolic model (Table 5), both diabetes duration and triglyceride levels remained significant predictors of CAP scores, with diabetes duration showing the greatest impact (β = 3.22, 95% CI: 1.93–4.51; *p* < 0.0001). This model explained 31.2% of CAP variability (R^2^ = 0.312, *p* < 0.0001).

In the model including IR indices (Table 6), METS-IR was positively associated with CAP (β = 1.55, 95% CI: 0.94–2.16, *p* < 0.0001), whereas eGDR showed a negative association (β = −7.74, 95% CI: −11.60 to −3.89, *p* = 0.0001). The model explained 34.0% of the variability in CAP values (R^2^ = 0.340, *p* < 0.0001).

**Figure 2:**
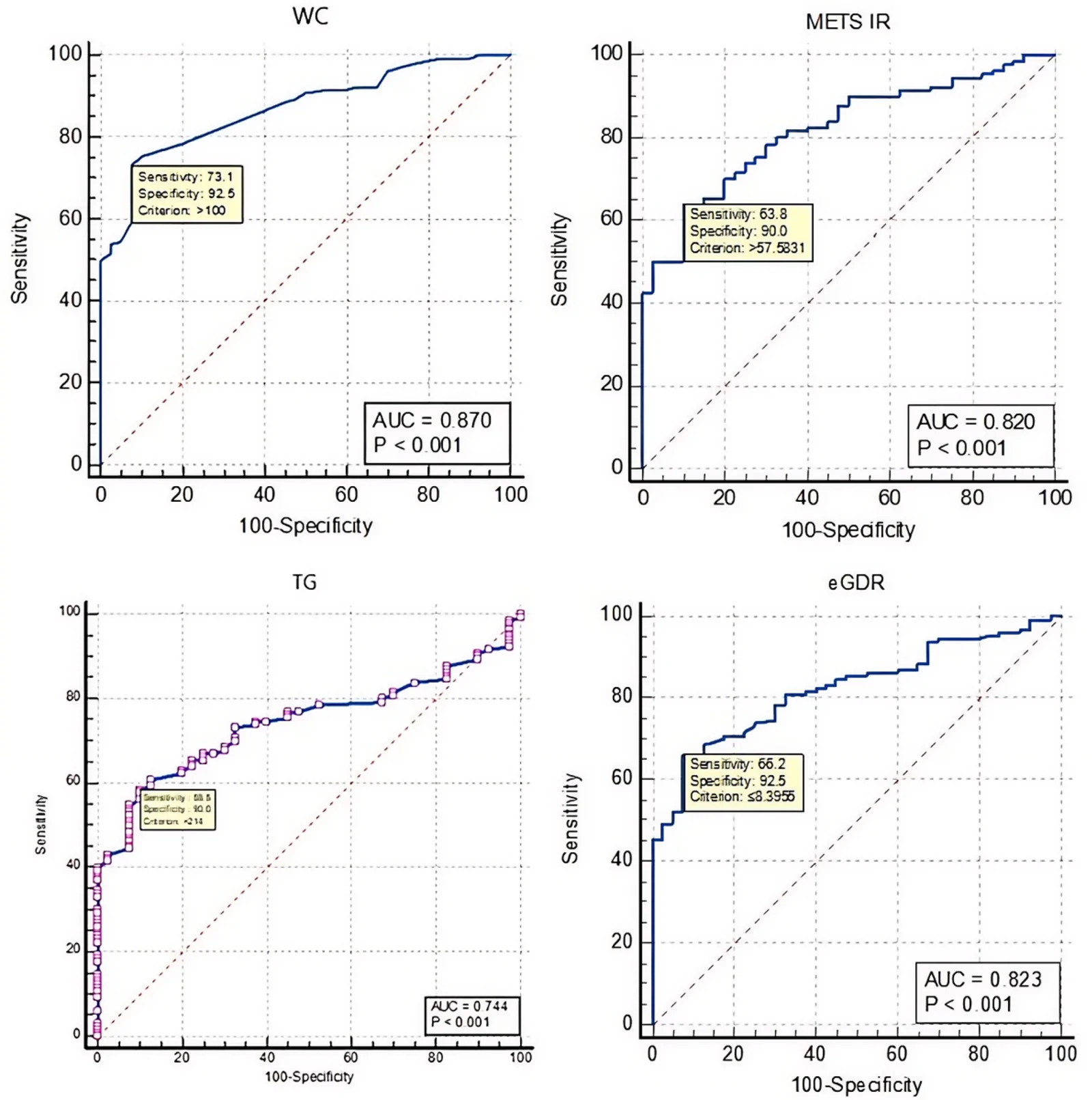
ROC curve analysis for identifying risk factors associated with metabolic dysfunction-associated steatotic liver disease. Abbreviations: WC, waist circumference; METS-IR, metabolic score for insulin resistance; TG, triglyceride; eGDR, estimated glucose disposal rate. The red dashed diagonal line represents the reference line corresponding to chance-level discrimination (AUC = 0.5).

Furthermore, ROC analysis was performed to better characterize risk factors associated with MASLD (Figure 2). Waist circumference proved the highest discriminatory ability in identifying MASLD patients (AUC = 0.870, 95% CI: 0.810–0.917, *p* < 0.0001), with a cut-off value of > 100 cm for predicting hepatic steatosis, showing a sensitivity of 73.1% and a specificity of 92.5%. Similarly, both METS-IR (AUC = 0.820, 95% CI: 0.754–0.875, *p* < 0.0001) and eGDR (AUC = 0.823, 95% CI: 0.758–0.878, *p* < 0.0001) demonstrated good discrimination. Triglyceride levels demonstrated moderate predictive performance (AUC = 0.744, 95% CI: 0.671–0.807, *p* < 0.0001), with values above 214 mg/dL associated with a sensitivity of 58.5% and specificity of 90.0%.

## 4. Discussion

The present study demonstrates that MASLD exacerbates the cardiometabolic risk profile in patients with type 2 diabetes. Furthermore, HbA1c, TyG, and METS-IR emerged as independent predictors of both MASLD and hepatic fibrosis severity, supporting their utility for noninvasive, early-stage risk stratification. The observed MASLD prevalence of 76.5% in our cohort stands at the higher end of previously reported estimates. This finding reinforces the importance of systematically recognizing MASLD as a major metabolic comorbidity in patients with T2DM [4].

Our findings also highlight the close association between MASLD and adverse metabolic features in T2DM. Individuals with MASLD had a longer duration of diabetes and significantly poorer glycemic control, as reflected by higher fasting plasma glucose, HbA1c, and C-peptide concentrations. These data are consistent with the central role of IR and chronic hyperglycemia in the pathogenesis of hepatic steatosis. Prolonged hyperglycemia may promote hepatic de novo lipogenesis, while compensatory hyperinsulinemia may enhance lipid accumulation within hepatocytes, thereby favoring the development and progression of MASLD [42]. Notably, diabetes duration showed the strongest correlation with CAP values (ρ = 0.578) and remained an independent predictor in multivariable analysis. This observation suggests that cumulative exposure to chronic metabolic dysfunction may contribute to progressive hepatic fat accumulation. Similar findings have been reported in other evaluated diabetic populations, in which longer diabetes duration has been associated with a higher risk of hepatic steatosis and fibrosis [43]. DM duration appears to be associated with MASLD development and progression, as results from cross-sectional and longitudinal studies support a clear temporal association between disease duration and liver disease severity [44]. In particular, a diabetes duration exceeding 10 years has been associated with more than a twofold increase in the odds of advanced liver fibrosis (OR = 2.06, 95% CI: 1.51–2.81), as assessed by the FIB-4 index, highlighting the cumulative impact of long-standing metabolic dysregulation on hepatic injury and fibrosis progression [45]. In our research, FIB-4 and liver stiffness values were low, potentially caused by the shorter DM duration of 5 years in our cohort, a fact that reflects the importance of cumulative exposure to associated metabolic risk factors.

In addition, patients with MASLD in our study also demonstrated a more atherogenic lipid profile, characterized by higher LDL-cholesterol and triglyceride levels, and lower HDL-cholesterol concentrations. Similar findings have been reported in previous studies [4,6,46]. In a cohort of 203 individuals with T2DM, MASLD was identified in 71.9% of participants, and multivariable analysis identified dyslipidemia, elevated LDL-cholesterol, increased HbA1c levels, and higher diastolic blood pressure as independent predictors of MASLD [4]. The concordance between our findings and prior studies further supports the view that MASLD in T2DM represents a hepatic manifestation of broader systemic metabolic dysfunction rather than an isolated liver disorder.

Our results also support the important role of dyslipidemia in the pathogenesis of MASLD. Among the evaluated lipid parameters, triglycerides showed one of the strongest correlations with CAP values and were independently associated with hepatic steatosis. In addition, patients with MASLD displayed a more atherogenic lipid profile, characterized by higher LDL-cholesterol and lower HDL-cholesterol levels compared with those without steatosis. This pattern is consistent with the dyslipidemia typically observed in insulin-resistant states and reflects the profound alterations in lipid metabolism that accompany hepatic fat accumulation [47]. Mechanistically, increased hepatic triglyceride synthesis, impaired lipid clearance, and enhanced very-low-density lipoprotein production contribute to the development of both dyslipidemia and steatosis, underscoring their shared metabolic origins [48]. Previous studies have similarly identified elevated triglycerides and reduced HDL-cholesterol as major metabolic correlates of MASLD and of greater cardiometabolic risk [49]. Taken together, these findings suggest that routine lipid parameters, particularly triglycerides, may help identify patients with T2DM at increased risk of MASLD.

Markers of adiposity also emerged as major determinants of hepatic steatosis in our cohort. Both BMI and waist circumference were significantly higher in patients with MASLD and remained independently associated with CAP values in multivariable models. Waist circumference showed the strongest association with CAP and the highest discriminatory performance for MASLD detection (AUC = 0.870). In addition, a threshold above 100 cm showed high specificity, supporting its potential value as a readily available clinical marker for identifying individuals at increased risk of hepatic steatosis, reinforcing the important role of central adiposity in MASLD pathogenesis. Even if WC > 100 cm had high specificity in our research, sensitivity was moderate-to-low, limiting its utility as a stand-alone screening parameter. Similar sensitivity results were obtained regarding TG. Still, these parameters maintain their clinical value in combination with other metabolic screening factors and the usage of validated noninvasive liver assessment tools. Visceral adipose tissue is metabolically active and contributes to increased portal free fatty acid delivery, systemic inflammation, and IR, all of which favor hepatic fat accumulation [50]. Accordingly, several studies have reported stronger associations between waist circumference and MASLD than between BMI and MASLD, highlighting the importance of fat distribution rather than total body weight in disease development [51].

Our results are further supported by longitudinal evidence demonstrating a temporal relationship between abdominal obesity and MASLD risk. In a population-based cohort with 16 years of follow-up, progressive increases in waist circumference were associated with a significantly higher incidence of MASLD, whereas reductions in waist circumference were linked to a lower risk of disease development [52]. Similarly, recent observational studies have consistently identified central obesity as one of the strongest predictors of both prevalent and incident MASLD [53]. Collectively, these findings suggest that waist circumference may serve not only as a marker of existing hepatic steatosis but also as a valuable tool for identifying individuals at risk of future disease progression, supporting its incorporation into routine metabolic risk assessment [54].

A key finding of our study was the strong correlation between hepatic steatosis and IR. Patients with MASLD had significantly higher METS-IR values and lower eGDR values compared with those without MASLD, and both indices remained independently associated with CAP in multivariable analysis. Moreover, ROC analysis demonstrated good discriminatory performance for both markers, supporting their potential utility as noninvasive tools for identifying hepatic steatosis in patients with T2DM. Our results are consistent with previous evidence supporting the use of surrogate insulin resistance indices in MASLD risk assessment [55]. METS-IR has been shown to correlate strongly with metabolic dysfunction and has emerged as a reliable predictor of hepatic steatosis across diverse populations [56]. Similarly, lower eGDR values, reflecting more severe IR, have been associated with adverse metabolic profiles and an increased likelihood of MASLD, particularly among individuals with diabetes [57]. The association of both METS-IR and eGDR with CAP in our cohort supports their clinical relevance. These indices may facilitate early identification of patients at increased risk of hepatic steatosis when more sophisticated diagnostic methods are unavailable. Used together, they may provide a broader assessment of the metabolic disturbances underlying MASLD than isolated biochemical parameters alone [58]. However, the insulin-resistance surrogate indices are biologically interrelated. As formal multicollinearity diagnostics were not available, the relative contribution and independent associations of predictors with CAP should be interpreted with caution. The consistency between our findings and those reported in large population-based cohorts, including recent epidemiological data from the UK Biobank, further supports their potential integration into clinical algorithms to improve MASLD detection and guide preventive interventions in high-risk individuals [59].

Regarding liver-related biomarkers, AST levels were significantly higher in patients with MASLD, whereas ALT levels showed no significant differences between groups. Although this pattern may reflect greater hepatometabolic impairment, transaminases alone are insufficient for accurate detection or staging of MASLD. Importantly, normal liver enzyme levels do not exclude clinically relevant hepatic steatosis, and reliance on transaminases alone may therefore lead to underrecognition of liver disease in patients with T2DM [60].

Our findings have clinical relevance, as waist circumference, triglycerides, METS-IR, and eGDR demonstrated good discriminatory performance for identifying patients with hepatic steatosis and may serve as simple, low-cost tools for risk stratification in routine DM management. Given the increasing burden of liver-related and cardiovascular complications associated with MASLD, incorporating these parameters into clinical risk assessment may facilitate earlier identification of patients who would benefit from further liver evaluation and closer cardiometabolic monitoring [61].

These results are consistent with previous studies showing that central obesity and hypertriglyceridemia are among the strongest metabolic predictors of MASLD, reflecting the close interplay between visceral adiposity, insulin resistance, and hepatic fat accumulation [62]. In particular, waist circumference has been repeatedly identified as a robust surrogate marker of visceral adiposity and an independent predictor of hepatic steatosis, often outperforming body mass index in risk stratification models [63]. Similarly, elevated triglyceride levels represent a central feature of the metabolic dysfunction underlying MASLD and have been consistently associated with both the presence and severity of hepatic steatosis [64].

The diagnostic performance of METS-IR observed in our study further supports accumulating evidence regarding its value as a noninvasive indicator of IR and broader metabolic dysfunction. Originally proposed by Bello-Chavolla et al., METS-IR has been shown to correlate closely with insulin sensitivity and to identify individuals at increased cardiometabolic risk [56]. More recent studies have also demonstrated significant associations between higher METS-IR values and the presence of MASLD, suggesting that this index may represent a practical alternative to more complex methods of assessing insulin resistance [65].

Similarly, lower eGDR values, reflecting more severe IR, were independently associated with hepatic steatosis in our cohort, an observation consistent with previous findings, particularly in diabetic populations. In these settings, lower eGDR has been linked to adverse metabolic profiles, hepatic fat accumulation, and increased cardiometabolic risk [66].

Several limitations of the present study should be recognized. First and foremost, the cross-sectional design precludes any causal inference between MASLD and the investigated metabolic variables. Therefore, the results should be interpreted as associative rather than causal. Second, the single-site design and relatively modest sample size of 170 patients, particularly the uneven distribution between the MASLD (*n* = 130) and non-MASLD (*n* = 40) groups, may limit the statistical power for robust multivariate analysis and affect the precision of the predictive cutoffs. Although we constructed separate parsimonious regression models by domain to mitigate the risk of overfitting and utilized the full cohort for continuous CAP analysis, the imbalance may still restrict the generalizability of the findings to broader populations with T2DM. Consequently, the results should be viewed as hypothesis-generating and require validation in larger, multi-center cohorts. Third, MASLD was diagnosed using CAP on transient elastography rather than liver biopsy, which remains the gold standard for assessing hepatic steatosis and steatohepatitis. The diagnosis method was chosen according to the study’s observational design and the absence of clinical indication for liver biopsy. Although CAP is a validated and widely used noninvasive method, the possibility of diagnostic misclassification cannot be completely excluded. Fourth, important lifestyle confounders, including dietary habits, physical activity, smoking status, and detailed alcohol consumption patterns, were not available in this retrospective cohort. Although patients with significant alcohol consumption were excluded based on established diagnostic criteria for MASLD, the lack of quantitative assessment for alcohol intake and other lifestyle factors limits our ability to fully adjust for their influence on hepatic steatosis and metabolic outcomes. These unmeasured variables may potentially confound the observed associations. Also, sex-related differences in anthropometric and metabolic characteristics may also be relevant to the interpretation of CAP, and a potential influence of sex cannot be excluded. Future studies should further evaluate sex-related differences in CAP and consider sex in the analysis of factors associated with CAP. Furthermore, as chronic low-grade inflammation is increasingly recognized as an important component of MASLD pathophysiology, the lack of inflammatory biomarkers or validated metabolic inflammation scores represents a further limitation. Thus, the potential contribution of metabolic inflammation could not be specifically assessed. Future prospective studies should incorporate such measures to further clarify the relationship between MASLD and metabolic inflammation. Finally, the influence of concurrent pharmacological therapies was not analyzed. Specifically, lipid-lowering agents, such as statins and fibrates, and glucose-lowering therapies, including pioglitazone and GLP-1 receptor agonists, may influence metabolic and hepatic parameters, including hepatic steatosis and liver enzyme levels. Additionally, the potential impact of antihypertensive medications was not assessed. The lack of adjustment for these medication classes represents a potential source of confounding bias, as they may independently influence the metabolic and hepatic parameters evaluated in this study.

Overall, our findings support the concept that MASLD in patients with T2DM is closely associated with obesity, IR, dyslipidemia, and longer diabetes duration. Moreover, the coexistence of diabetes, obesity, and MASLD may be associated with an increased long-term risk of hepatocellular carcinoma, further emphasizing the importance of systematic assessment of these interrelated metabolic factors in routine clinical practice may facilitate earlier identification of high-risk individuals and help reduce the burden of both hepatic and cardiometabolic complications.

## 5. Conclusions

Our findings indicate that patients with MASLD exhibit a less favorable metabolic profile than those without MASLD, highlighting the importance of early identification of high-risk individuals at increased metabolic and hepatic risk and the implementation of comprehensive therapeutic strategies targeting glycemic control and cardiometabolic risk. Higher HbA1c and insulin resistance indices, including TyG and METS-IR, were associated with the presence of MASLD and with higher noninvasive indices of hepatic steatosis and fibrosis. Although HbA1c remains a widely used and easily accessible marker in routine clinical practice, TyG and METS-IR may provide additional information for metabolic and hepatic risk stratification. Consequently, combined assessment of these parameters may therefore contribute to the early recognition of individuals at higher risk who may benefit from further hepatic evaluation and targeted cardiometabolic management. These findings should be interpreted in the context of the non-invasive assessment of hepatic steatosis and fibrosis used in the present study and require confirmation in prospective studies using histological assessment where appropriate.

## Figures and Tables

**Table 1:** Clinical and Biochemical Characteristics of the Study Population, Compared by Gender.

Variable	Overall (*n* = 170)	Men (*n* = 81)	Women (*n* = 89)	*p-*Value
Age (years)	61 (56, 67)	62 (54.7, 66.2)	61 (56, 67)	0.977
DM duration (years)	5 (1, 10)	4 (1, 9)	6 (1, 10)	0.279
Weight (kg)	98 (88, 112)	100 (89, 118)	97 (86, 109.3)	**0.039**
BMI (kg/m^2^)	35.5 (30.9, 39.9)	36.4 (31.4, 40.9)	33.9 (30.8, 38.3)	**0.029**
WC (cm)	110 (98, 121)	114 (98, 125.2)	100 (96.7, 118)	**0.005**
Glycemia (mg/dL)	131 (111, 141)	125 (110, 139.2)	135 (115.7, 145)	**0.045**
HbA1c (%)	7.2 (6.8, 8)	7.1 (6.7, 8)	7.4 (6.87, 8)	0.189
C-peptide (ng/mL)	4.03 (2.8, 5.1)	4 (2.7, 5.1)	4.2 (2.84, 5.12)	0.738
LDL-C (mg/dL)	99 (88, 120)	100 (88, 121)	99 (88, 120)	0.930
HDL-C (mg/dL)	41.6 (36.1, 45)	42 (36, 46.1)	41 (36.3, 45)	0.462
TG (mg/dL)	211 (143, 287)	214 (144, 301.2)	199 (138.7, 258)	0.128
AST (U/L)	22 (18.9, 27.8)	21.4 (18.3, 27)	22.6 (19.2, 27.8)	0.192
ALT (U/L)	24 (18, 36)	24 (18, 36.2)	24 (17.9, 35.2)	0.996
MET-IR	58.2 (49.6, 68.1)	61.1 (51.1, 70.1)	56.2 (49.3, 66.7)	0.109
eGDR	8.14 (6.52, 9.29)	7.49 (6.22, 9.12)	8.47 (6.85, 9.31)	0.117
Tg/HDLc	4.83 (3.27, 7.52)	4.91 (3.44, 7.98)	4.69 (3.02, 6.76)	0.446
TyG index	5.08 (4.90, 5.24)	5.09 (4.91, 5.26)	5.06 (4.88, 5.22)	0.584
CAP (Db/m)	289.5 (243, 304)	294 (245, 310.2)	288 (232.2, 301)	0.350
Liver Fibrosis (kPa)	8.20 (5.70, 12)	8.10 (5.85, 11.5)	8.40 (5.70, 12)	0.839
FIB-4	1.123 (0.86, 1.42)	1.04 (0.79, 1.41)	1.20 (0.94, 1.42)	0.119
APRI	0.282 (0.23, 0.38)	0.26 (0.21, 0.37)	0.30 (0.24, 0.40)	0.162

Data are represented as the median (interquartile range, IQR) or as percentages (%; *n*). Abbreviations: DM, diabetes mellitus, BMI, body mass index; WC, waist circumference; HbA1c, glycated hemoglobin; LDL-C, low-density lipoprotein cholesterol; HDL-C, high-density lipoprotein cholesterol; TG, triglyceride; AST, aspartate aminotransferase (U/L); ALT, alanine aminotransferase (U/L); METS-IR, metabolic score for insulin resistance; eGDR, estimated glucose disposal rate; Tg/HDLc, triglyceride to high-density lipoprotein cholesterol ratio; TyG index, triglyceride–glucose index; CP2 index, C-peptide index 2; CAP, controlled attenuation parameter; FIB-4, fibrosis-4 index; APRI, aspartate aminotransferase to platelet ratio index. Statistically meaningful differences, with a probability value of *p* < 0.05, are represented in bold.

**Table 2:** Comparison of the studied patients’ parameters by the presence of MASLD.

Variable	Without MASLD (*n* = 40)	with MASLD (*n* = 130)	*p*-Value
Males (%)	17 (42.5)	64 (49.2)	0.457 ^χ2^
Age (years)	60 (54, 66)	61.5 (56, 67)	0.273 ^U^
Weight (kg)	89 (86, 96.5)	104 (90, 118)	**<0.000 ^U^**
WC (cm)	95.5 (88.5, 98)	114.5 (100, 125)	**<0.000 ^U^**
BMI (kg/m^2^)	30.8 (28.1, 33.9)	36.92 (32.4, 41.03)	**<0.0001 ^U^**
DM duration (years)	4.00 (1.00, 6.50)	5.00 (1.00, 12.0)	**0.043 ^U^**
Glycaemia (mg/dL)	121.5 (111, 133)	134.5 (116, 144)	**0.009 ^U^**
HbA1c (%)	7.00 (6.80, 7.40)	7.40 (6.90, 8.00)	**0.006 ^U^**
C-peptide (ng/mL)	3.00 (2.00, 4.00)	4.39 (3.27, 5.30)	**<0.000 ^U^**
LDL-C (mg/dL)	95 (87.5, 100)	101.5 (90,124)	**0.003 ^U^**
HDL-C (mg/dL)	44.3 (40.5, 46.4)	40 (34.3, 45.0)	**0.0004 ^U^**
TG (mg/dL)	144 (133, 181)	239 (154, 298)	**<0.0001 ^U^**
AST (U/L)	19.65 (16, 22.8)	22.8 (19.3, 28)	**0.003 ^U^**
ALT (U/L)	24.5 (18.6 to 34)	24.0 (18.0, 36.0)	0.867 ^U^
METS-IR	47.86 (44.1, 53.6)	62.02 (52.9,71.2)	**<0.000 ^U^**
eGDR	9.45 (8.58, 11.98)	7.35 (6.25, 8.59)	**<0.000 ^U^**

^U^ Mann–Whitney test and ^χ2^ chi-squared. Data are expressed as the median (interquartile range, IQR) or percentage (*n*, %). Abbreviations: BMI, body mass index; DM, diabetes mellitus; HbA1c, glycated hemoglobin; LDL-C, low-density lipoprotein cholesterol; HDL-C, high-density lipoprotein cholesterol; TG, triglycerides; AST, aspartate aminotransferase; ALT, alanine aminotransferase; METS-IR, metabolic score for insulin resistance; eGDR, estimated glucose disposal rate. Statistically significant differences, with a probability value of *p* < 0.05, are represented in bold.

**Table 3:** Correlation Analysis of CAP with Anthropometric, Lipid, and Metabolic Parameters.

	BMI (kg/m^2^)	WC (cm)	DM Duration (years)	LDL-c (mg/dL)	TG (mg/dL)	HDL-c (mg/dL)	TG/ HDL-c Ratio	TyG Index	METS-IR	eGDR
**ρ**	0.291	0.237	0.578	−0.258	0.403	−0.155	0.362	0.367	0.325	−0.234
***p*** **-value**	**0.0008**	**0.0067**	**<0.0001**	**0.0030**	**<0.0001**	**0.0777**	**<0.0001**	**<0.0001**	**0.0002**	**0.0073**

Spearman correlation test. Abbreviations: CAP, controlled attenuation parameter; ρ, Spearman rank correlation coefficient; BMI, body mass index; WC, waist circumference; DM, diabetes mellitus; LDL-c, low-density lipoprotein cholesterol; TG, triglyceride; HDL-c, high-density lipoprotein cholesterol; TyG index, triglyceride–glucose index; METS-IR, metabolic score for insulin resistance; eGDR, estimated glucose disposal rate. Statistically significant values (*p* < 0.05) are shown in bold.

**Table 4:** Multiple linear regression analysis for predictors of CAP in the entire cohort.

Variable	β Coefficient	S.E.	95% CI	t	Semipartial r	*p*-Value
(Constant)	−28.27	34.00	−95.41–38.86	−0.83		0.4069
Age	1.00	0.3869	0.24–1.76	2.58	0.15	**0.0106**
BMI	1.83	0.2694	0.58–3.08	5.91	0.36	**0.0042**
WC	1.59	0.6312	1.06–2.13	2.89	0.17	**<0.0001**

Abbreviations: BMI, body mass index; WC, waist circumference. Statistically significant differences, with a probability value of *p* < 0.05, are represented in bold. Notes: Coefficient of determination R2 = 0.3756, R2-adjusted = 0.3644, multiple correlation coefficient = 0.6129, residual standard deviation = 46.2192, F-ratio = 33.29150.

**Table 5:** Multiple linear regression analysis for CAP with diabetes duration and triglyceride levels.

Independent Variables	β Coefficient	S.E.	95% CI	t	Semipartial r	*p*-Value
(Constant)	191.67	10.61	170.71–212.62	18.05		**<0.0001**
Diabetes duration	3.21	0.65	1.92–4.50	4.92	0.31	**<0.0001**
TG	0.27	0.04	0.18–0.37	5.97	0.38	**<0.0001**

Abbreviations: TG = triglycerides. Statistically significant differences, with a probability value of *p* < 0.05, are represented in bold. Notes: Coefficient of determination R2 = 0.3121, R2-adjusted = 0.3038, multiple correlation coefficient = 0.5586, residual standard deviation = 48.3696, F-ratio = 37.87992.

**Table 6:** Multiple linear regression analysis for CAP with insulin resistance indices.

Independent Variables	β Coefficient	S.E.	95% CI	t	Semipartial r	*p*-Value
(Constant)	242.88	30.59	182.48–303.29	7.93		**<0.0001**
METS-IR	1.55	0.31	0.93–2.16	4.98	0.31	**<0.0001**
eGDR	−7.74	1.95	−11.59–−3.89	−3.96	0.24	**0.0001**

Abbreviations: METS-IR, metabolic score for insulin resistance; eGDR, estimated glucose disposal rate. Statistically significant differences, with a probability value of *p* < 0.05, are represented in bold. Notes: Coefficient of determination R2 = 0.3401, R2-adjusted = 0.3322, multiple correlation coefficient = 0.5832, residual standard deviation = 47.3750, F-ratio = 43.03015.

## Data Availability

The data presented in this study are available on request from the corresponding author. The data are not publicly available due to local privacy and data protection regulations.

## Abbreviations

The following abbreviations are used in this manuscript:

DM	Diabetes mellitus
T2DM	Type 2 diabetes mellitus
MASLD	Metabolic dysfunction associated steatotic liver disease
IR	Insulin resistance
CKD	Chronic kidney disease
PN	Peripheral neuropathy
DR	Diabetic retinopathy
HF	Heart failure
IHD	Ischemic heart disease
HTN	Hypertension
AF	Advanced fibrosis
BMI	Body mass index
WC	Waist circumference
HbA1c	Glycated hemoglobin
LDL-C	Low-density lipoprotein cholesterol
HDL-C	High-density lipoprotein cholesterol
TG	Triglyceride
AST	Aspartate aminotransferase (U/L)
ALT	Alanine aminotransferase (U/L)
METS-IR	Metabolic score for insulin resistance
eGDR	Estimated glucose disposal rate
Tg/HDLc	Triglyceride to high-density lipoprotein cholesterol ratio
TyG index	Triglyceride–glucose index
CP2 index	C-peptide index 2
CAP	Controlled attenuation parameter
FIB-4	Fibro-sis-4 index
APRI	Aspartate aminotransferase to platelet ratio index
